# Distribution characteristics of initial lesion sites for diabetic foot ulcers in China: a national multicenter survey

**DOI:** 10.3389/fendo.2026.1983798

**Published:** 2026-10-21

**Authors:** Yingzhou Shi, Tao Xue, Liren Ma, Lijun Zou, Kexin Li, Yanxiang Wu, Qiuhong Zhou, Yanmei Yu, Xiaorui Jiang, Lin Li, Jingyi Zhang, Huifeng Zhang, Penghua Wang, Bingquan Yang, Hong Zhu, Aihong Wang, Yufeng Jiang, Zhangrong Xu, Xingwu Ran, Qiu Li

**Affiliations:** 1 Key Laboratory of Endocrine Glucose & Lipids Metabolism and Brain Aging, Ministry of Education, Department of Endocrinology, Shandong Provincial Hospital Affiliated to Shandong First Medical University, Jinan, Shandong, China; 2 Department of Endocrinology, Jiading District Central Hospital Affiliated Shanghai University of Medicine & Health Sciences, Shanghai, China; 3 Department of Peripheral Vascular Disease, Pingdingshan Traditional Chinese Medicine Hospital, Pingdingshan, Henan, China; 4 Department of Wound Repair Surgery, Liyuan Hospital, Tongji Medical College, Huazhong University of Science and Technology, Wuhan, Hubei, China; 5 Department of Endocrinology, Jilin Province People’s Hospital, Changchun, China; 6 Department of Endocrinology, Air Force Hospital of Northern Theater Command of PLA, Shenyang, China; 7 Teaching and Research Section of Clinical Nursing, Xiangya Hospital of Central South University, Changsha, Hunan, China; 8 National Clinical Research Center for Geriatric Diseases, Xiangya Hospital of Central South University, Changsha, Hunan, China; 9 Department of Endocrinology, Mudanjiang Cardiovascular Hospital, Mudanjiang, China; 10 Department of Endocrinology and Foot & Ankle Surgery, Yantai Yuhuangding Hospital, Qingdao University, Yantai, Shandong, China; 11 Department of Endocrinology, Sir Run Run Shaw Hospital, School of Medicine, Zhejiang University, Hangzhou, China; 12 Department of Endocrinology, Kailuan General Hospital Affiliated to North China University of Science and Technology, Tangshan, Hebei, China; 13 Department of Endocrinology and Metabolism, Henan Provincial People’s Hospital, People’s Hospital of Zhengzhou University, Zhengzhou, Henan, China; 14 Department of Diabetic Foot, NHC Key Laboratory of Hormones and Development, Tianjin Key Laboratory of Metabolic Diseases, Chu Hsien-I Memorial Hospital & Tianjin Institute of Endocrinology, Tianjin Medical University, Tianjin, China; 15 Department of Endocrinology, Zhongda Hospital, Institute of Diabetes, School of Medicine, Southeast University, Nanjing, China; 16 Department of Endocrinology, The First Affiliated Hospital of Wenzhou Medical University, Wenzhou, Zhejiang, China; 17 Department of Endocrinology, The Ninth Medical Center, Chinese PLA General Hospital, Beijing, China; 18 Department of Tissue Repair and Regeneration, Chinese PLA General Hospital, Beijing, China; 19 Department of Endocrinology and Metabolism, Innovation Center for Wound Repair, Diabetic Foot Care Center, West China Hospital, Sichuan University, Chengdu, Sichuan, China

**Keywords:** diabetic foot ulcer, diabetic peripheral neuropathy, lower extremity arterial disease, precipitating factors, ulcer location

## Abstract

**Aims:**

Systematic data on initial diabetic foot ulcer (DFU) locations in China remain limited. We investigated the distribution of initial DFU sites and precipitating factors, together with secondary associations of recorded anatomical site involvement with diabetic peripheral neuropathy (DPN) and lower extremity arterial disease (LEAD).

**Methods:**

This multicenter cross-sectional analysis used an analytical database containing 3,105 records from 2,892 patients recruited at 77 Chinese hospitals (October 2020-April 2022). The earliest dated record was selected for each patient. Physicians recorded, using a 56-region foot classification, the sites where the ulcer first appeared (7,813 site entries from 2,892 patients). Secondary patient-level associations were estimated using age- and sex-adjusted logistic regression with hospital-cluster-robust standard errors.

**Results:**

Among 2,892 patients, the most frequent initial-site categories were between toes (1,102/2,892, 38.1%), dorsum of toe (883/2,892, 30.5%), and toe tip (770/2,892, 26.6%). In secondary analyses, right-foot sole involvement was more frequent than left-foot involvement (5.91% vs 4.32%; FDR-adjusted q=0.034), whereas right-foot toe-tip involvement was less frequent (11.89% vs 14.80%; q=0.022). Leading precipitating factors were accidental injuries (32.2%), footwear-related injury (25.4%), and unidentified causes (16.0%). LEAD alone was associated with dorsal-toe involvement (OR = 1.71, 95% CI 1.30-2.24; q=0.002), while DPN alone was associated with heel involvement (OR = 1.83, 95% CI 1.22-2.76; q=0.015).

**Conclusions:**

This multicenter cross-sectional study characterizes the distribution of initial DFU sites and precipitating factors in China and identifies secondary site-specific neurovascular associations that may inform targeted prevention.

## 1 Introduction

Diabetes mellitus is a highly prevalent chronic metabolic disease worldwide, and its complications have become important factors threatening patients’ life and health (1). Among these, diabetic foot disease (DFD) is one of the most severe chronic complications of diabetes, primarily manifesting as foot infections, ulcerations, and deep tissue destruction, accompanied by high rates of disability and mortality (2, 3). A national multicenter cohort study revealed that the annual incidence of foot ulcers among Chinese diabetic patients was 8.1%, with an amputation rate of 5.1% among diabetic foot ulcer patients (4), which is significantly higher than that in European and American countries. Simultaneously, diabetic foot disease imposes a heavy burden on healthcare systems. A survey of 19 urban central hospitals in China showed that the average medical cost reached USD 5,932 (approximately 38,000 RMB), which was 44.6% higher than that of non-diabetic amputation patients, creating enormous economic pressure on patients’ families and society (5).

The initial location of diabetic foot disease refers to the anatomical site where foot pathology first occurs. Different initial locations may be closely related to neuropathy (such as sensory loss) or vascular disease (such as insufficient blood supply) (6). Studying the distribution characteristics of initial locations helps identify high-risk areas in different patients, thereby guiding individualized targeted prevention measures. For example, protective care for areas prone to ulceration, local pressure reduction, or interventions to improve blood supply can significantly reduce the risk of diabetic foot disease development. On the other hand, the initial precipitating factors of diabetic foot disease refer to external factors that trigger pathological changes, such as trauma, inappropriate friction from shoes and socks, burns, or other physical injuries. These external factors can also be avoided through reasonable intervention measures. For instance, selecting appropriate footwear, avoiding barefoot walking, or strengthening foot protection can effectively reduce the occurrence of initial precipitating factors. Therefore, studying the characteristics of initial locations and precipitating factors is of great significance for early prevention and control of diabetic foot disease.

Currently, there is a lack of systematic research on the initial locations and precipitating injury causes of diabetic foot disease, particularly large-sample multicenter studies targeting the Chinese population. Although the International Working Group on the Diabetic Foot (IWGDF) provides guidelines for diabetic foot prevention and management (7), these guidelines are primarily based on research data from European and American populations and lack targeted consideration of the characteristics of Asian populations, particularly the Chinese population. Existing studies have shown significant differences in foot anatomical structure, walking habits, footwear choices, and foot pressure distribution among different ethnic and regional populations (8, 9). These differences may lead to variations in the pathogenic patterns of diabetic foot ulcers.

Addressing the aforementioned research gaps, this study employed a 56-region foot classification system that captures detailed anatomical regions and can be aggregated into nine site groups, and analyzed 2,892 patients. The primary objective was to describe the distribution of initial diabetic foot ulcer sites and of precipitating factors in this hospital-based sample. The secondary objectives were to compare left and right feet and to examine associations of diabetic peripheral neuropathy (DPN) and lower extremity arterial disease (LEAD) with recorded anatomical site involvement. These analyses provide evidence for developing diabetic foot prevention strategies tailored to the Chinese population and have clinical and public health implications for reducing diabetic foot ulceration and its burden.

## 2 Materials and methods

### 2.1 Study subjects

We utilized the WeChat public account “Diabetic Foot Wound Survey” as a research tool to gather data across 77 hospitals in 14 provincial-level regions. The analytical database comprised 3,105 records from 2,892 patients. For patient-level analyses, one index record was selected for each patient using the earliest Entry date, yielding 2,892 patient records, which contained 7,813 recorded site entries.

The study period was set from October 2020 to April 2022. The included patients met the diagnostic criteria for diabetes and diabetic foot disease based on the World Health Organization 1999 criteria (10) and the International Working Group on the Diabetic Foot 2023 definitions (11). Eight exact duplicate records and four records without a recorded ulcer site were excluded. For patients with more than one record, the earliest record was analyzed.

Participating hospitals were recruited through provincial diabetic foot alliances using non-probability convenience sampling. Each provincial diabetic foot alliance was designated as a survey region, with a representative diabetic foot physician selected from each region’s main alliance institution or a physician with regional leadership status. These representatives needed extensive experience with diabetic foot specialty teams in tertiary grade A hospitals. They led data collection for diabetic foot patients in hospitals within their jurisdiction, including community specialty hospitals. Data could come from outpatient departments and wards with diabetic foot treatment capabilities (including endocrinology, hand and foot surgery, burn departments, dermatology, etc.) and community health centers. The information was ultimately uploaded to the platform. This study was approved by the Ethics Committee for Biomedical Research Involving Human Subjects of Shandong Provincial Hospital.

### 2.2 Recording basic variables of surveyed patients

Data collection included (1): detailed medical history, including age, gender, occupation, place of residence (urban or rural), smoking history, frequency of onset, duration of diabetes, and duration of diabetic foot disease (2); assessment of diabetic peripheral neuropathy (DPN) and/or lower extremity arterial disease (LEAD) using relevant auxiliary examination tools; (3) detailed inquiry about foot disease history and foot examination to identify the external cause of injury and initial site of diabetic foot ulcers (DFU); and (4) secondary analysis of recorded anatomical site involvement according to DPN, LEAD, and external injury cause.

### 2.3 Foot zoning

To ensure consistent anatomical coding, we divided the foot into 56 regions based on anatomical structure and possible trauma locations. Participating physicians were trained in the use of the survey and the region definitions and selected the affected regions from the 56 regions after examining the foot. As shown in **Figure 1** and **Supplementary Table S1**, positions were named according to their anatomical locations. For each patient, the earliest dated record was selected. As multiple anatomical sites could be recorded for each patient, all sites entered in the left- and right-foot fields were regarded as initial sites and analyzed as non-mutually exclusive patient-level indicators. Overall, 1,354 patients (46.8%) had multiple recorded sites, and 20 (0.7%) had bilateral involvement. The 56 positions were grouped into eight prespecified anatomical areas—sole of foot, dorsum of toe, dorsum of foot, heel, between toes, subungual toe, toe tip, and ankle—plus an “Other” category for locations not captured by these definitions.

**Figure 1:**
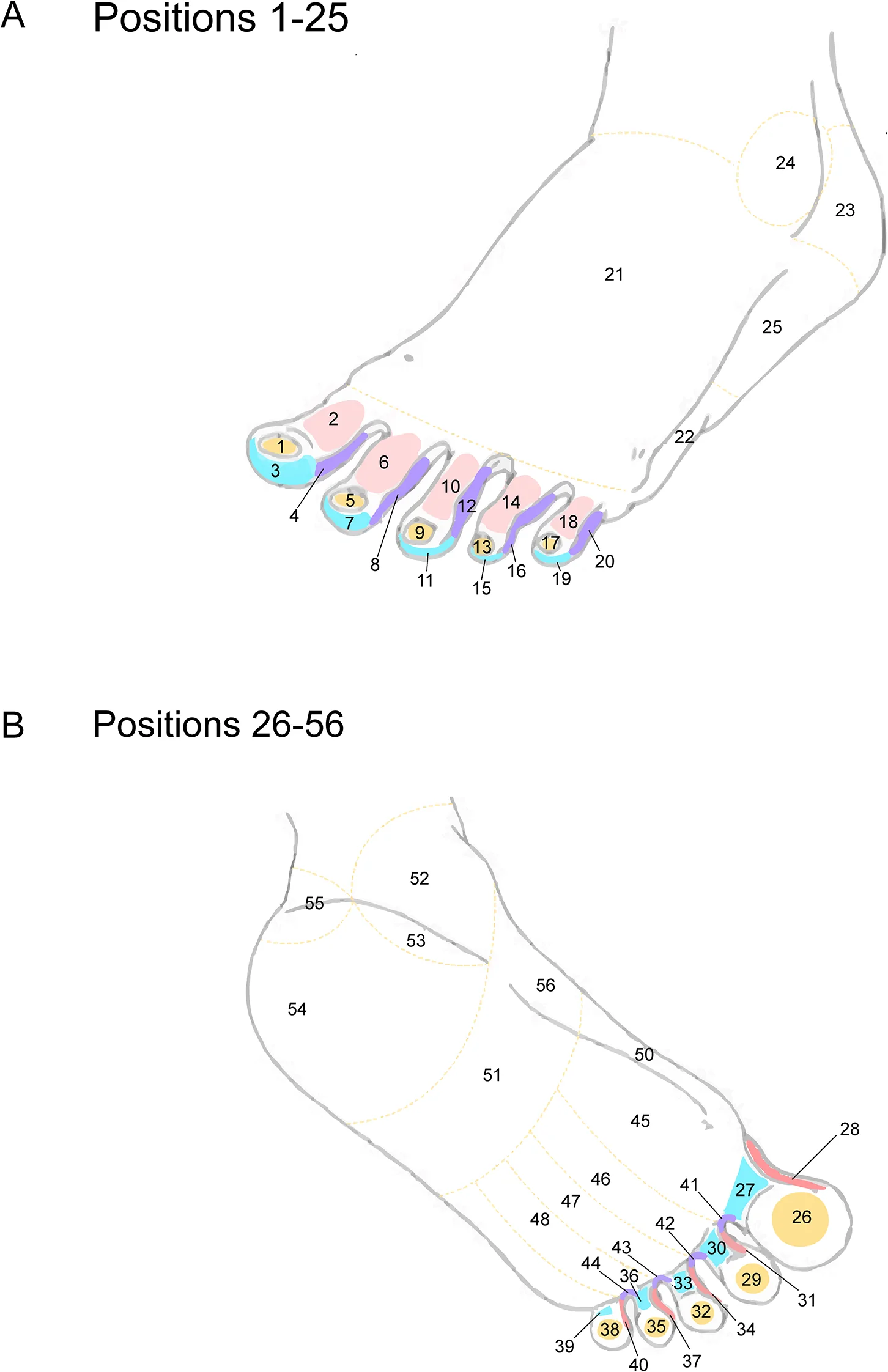
Fifty-six-region anatomical map used to code the initial diabetic foot ulcer site. **(A)** Dorsal view, positions 1–25. **(B)** Plantar view, positions 26–56. Each number denotes one region. Numbered region names and their mapping to the nine grouped anatomical categories are given in **Supplementary Table S1**.

### 2.4 Injury triggers and their classification

This study compiled 62 different external injury origins, summarizing them into 5 major categories. If the cause included “abrasion from shoes,” it was classified as “footwear-related injury.” If the cause included “mosquito/insect bite,” “nail puncture,” “crushing,” “bumping,” “scalding,” “pressure breakage,” “burning,” “abrasion,” “bleeding point,” “donkey stepping,” “friction breakage,” “sprain,” or “impact injury,” it was classified as “accidental injury.” If the cause included “walking barefoot,” “toenail cutting injury,” “foot soaking,” “puncture by foreign object,” “acupuncture,” “applying ointment,” “tearing,” “scratching,” “self-removal of callus,” “callus cutting injury,” “heat therapy,” “manual removal of dead skin,” “ulceration after applying plaster,” “small needle knife,” “callus trimming leading to ulceration,” “self-peeling skin,” “self-extraction of nails,” “self-cutting calluses,” “self-removal of corns,” “self-tearing skin,” “self-tearing,” or “self-grooming feet,” it was classified as “personal behavior.”

Skin lesions and infections included ischemic or vascular lesions, frostbite, spontaneous blisters, callus-related infection or ulceration, edema, dry fissuring or pruritic excoriation, corns, interdigital maceration, periungual or subungual infections, fungal infection, gout, gangrene, and other maceration. Causes recorded as “unknown” or without a specified external cause were classified as “unidentified.” The five categories were derived from the recorded free-text causes through consensus discussion among several diabetic foot specialists.

### 2.5 Diagnostic criteria for DPN and LEAD

The diagnostic criteria for DPN were: (1) Patients with clinical symptoms such as lower limb pain, paresthesia, numbness, and abnormalities in any one of ankle reflexes, pressure touch, vibration sensation, needle pain sensation, or temperature sensation; (2) For those without clinical symptoms, abnormalities in any two of the above five examinations; (3) Confirmed lower limb neuropathy by electromyography, excluding non-diabetic causes.

The diagnostic criteria for LEAD were: (1) Typical clinical manifestations of lower limb arterial stenosis or occlusion, such as intermittent claudication, rest pain, foot gangrene, etc.; (2) Resting ankle-brachial index (ABI) ≤0.90, or ≥1.3 regardless of whether the patient had lower limb discomfort symptoms; (3) Arterial lower limb vascular ultrasound, Doppler vascular imaging, CT vascular imaging, magnetic resonance vascular imaging, or lower limb angiography indicating occlusive lesions in at least one lower limb arterial vessel or stenosis greater than 50% in above-knee arterial vessels. DPN and LEAD were diagnosed by the treating physicians according to the criteria above.

### 2.6 Statistical analysis methods

All analyses were performed in R. Continuous variables are summarized as mean (standard deviation), and categorical variables as n (%), using available observations for each variable. For the initial-site distribution and for laterality, neurovascular, and trigger-by-site analyses, all locations listed in the index record were converted to binary patient-level site-involvement indicators, which were non-exclusive across anatomical categories. For supplementary 56-region descriptions, locations separated by Chinese or English commas or semicolons were treated as distinct location points, with all location points used as the denominator.

For the secondary recorded-site analyses, left-versus-right site involvement was compared within patients using McNemar tests. For each anatomical site other than “Other”, associations between neurovascular status and recorded site involvement were estimated using patient-level logistic regression adjusted for age and sex. Age and sex were prespecified as the basic demographic adjustment variables. In a sensitivity analysis, models were additionally adjusted for smoking and diabetes duration. The reference category was no DPN and no LEAD. Hospital-cluster-robust standard errors were used to account for within-hospital correlation. These models used complete cases for site outcome, neurovascular status, age, sex, and hospital. Missing data were not imputed. Descriptive statistics used all available observations for each variable.

All tests were two-sided. For each of the nine grouped anatomical sites, the association between trigger category and patient-level site involvement was assessed using a Pearson χ² test. Benjamini-Hochberg false-discovery-rate correction was applied separately across the nine trigger-by-site global tests, the nine left-versus-right comparisons, and the 24 neurovascular coefficients in the secondary neurovascular model. P<0.05 and FDR-adjusted q<0.05 were reported, with q values used to identify results robust to multiple testing.

## 3 Results

### 3.1 General patient information

The analytical database comprised 3,105 records from 2,892 patients, and patient-level descriptive analyses used one index record per patient (n=2,892). Participant characteristics are summarized in **Table 1**. The mean age was 63.4 ± 12.3 years, and 66.6% of participants with known sex were male (1,888/2,835). Among those with known smoking status, 31.0% (826/2,665) reported smoking. Retired or unemployed individuals accounted for 39.0% (1,129/2,892), farmers for 27.8% (804/2,892), and employed individuals for 13.5% (389/2,892). Among participants with known residence, 61.1% (1,642/2,689) lived in urban areas and 38.9% (1,047/2,689) in rural areas. Mean diabetes duration was 12.9 ± 8.6 years and mean DFU duration was 94.2 ± 187.1 days. Among 2,826 participants with known neurovascular status, 49.6% (1,403) had both DPN and LEAD, 24.1% (682) had DPN only, 10.7% (302) had LEAD only, and 15.5% (439) had neither.

**Table 1:** Participant characteristics.

Characteristic	Summary
Age, years	63.4 (12.3)
Sex	
Male	1,888 (66.6%)
Smoking history	
Yes	826 (31.0%)
Occupation	
Employed	389 (13.5%)
Farmer	804 (27.8%)
Retired or unemployed	1,129 (39.0%)
Unknown	570 (19.7%)
Residence	
Rural	1,047 (38.9%)
Urban	1,642 (61.1%)
Diabetes duration, years	12.9 (8.6)
Diabetic foot ulcer duration, days	94.2 (187.1)
Neurovascular status	
Neither DPN nor LEAD	439 (15.5%)
DPN only	682 (24.1%)
LEAD only	302 (10.7%)
DPN and LEAD	1,403 (49.6%)

Values are mean (SD) for continuous variables and n (%) for categorical variables. Percentages are calculated using available observations. DPN, diabetic peripheral neuropathy; LEAD, lower extremity arterial disease.

### 3.2 Overall distribution of initial onset sites

Among the 2,892 patients, between toes was the most frequently involved initial-site category (1,102/2,892, 38.1%), followed by dorsum of toe (883/2,892, 30.5%), toe tip (770/2,892, 26.6%), Other (727/2,892, 25.1%), subungual toe (615/2,892, 21.3%), dorsum of foot (336/2,892, 11.6%), heel (304/2,892, 10.5%), sole of foot (296/2,892, 10.2%), and ankle (162/2,892, 5.6%). Because patients could have more than one recorded site, these categories were not mutually exclusive and did not sum to 100% (**Figure 2**). The Other category comprised plantar toe pads (375 patients), plantar interphalangeal-joint regions (305), lateral metatarsal or midfoot regions (174), and medial metatarsal or midfoot regions (108); all component regions are listed in **Supplementary Table S1**. In the supplementary 56-region description, 2,892 patients contributed 7,813 detailed location points. The most frequent detailed locations were the first-toe dorsum (378/7,813, 4.8%) and the first-toe tip (376/7,813, 4.8%). The 1,464 nonempty left-foot cells contributed 3,999 points, and the 1,448 nonempty right-foot cells contributed 3,814 points (**Supplementary Figure S1**).

**Figure 2:**
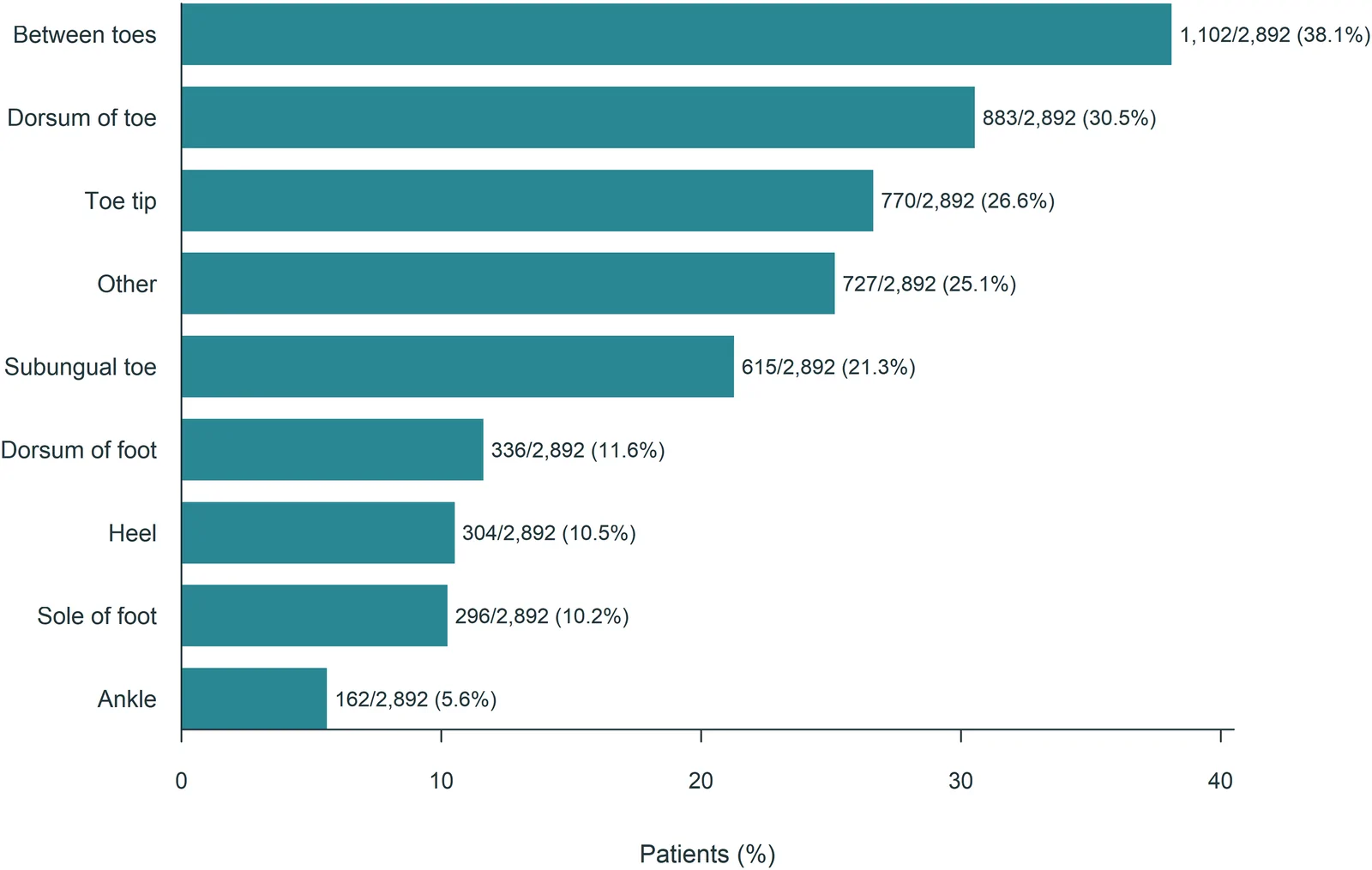
Distribution of initial diabetic foot ulcer locations among 2,892 patients. The earliest dated record was selected for each patient. Bars show n/N and percentage.

### 3.3 Analysis of ulcer triggers

Among all 2,892 patients, accidental injuries accounted for 32.2% (932), footwear-related injury for 25.4% (736), unidentified causes for 16.0% (462), personal behaviors for 15.3% (442), and skin lesions and infections for 11.1% (320). Trigger category was associated with eight of the nine grouped anatomical sites after FDR correction, with heel as the sole exception (**Supplementary Figure S2**). The unidentified category comprised causes recorded as unknown or without a specified external cause.

### 3.4 Differences in recorded anatomical site involvement between left and right feet

In paired patient-level comparisons, right-foot sole involvement was more frequent than left-foot involvement (5.91% vs 4.32%; right-minus-left difference, 1.59 percentage points; McNemar P = 0.008; FDR-adjusted q=0.034). Conversely, right-foot toe-tip involvement was less frequent than left-foot involvement (11.89% vs 14.80%; difference, −2.90 percentage points; P = 0.002; q=0.022). No other grouped site showed a side difference after FDR correction (**Figure 3**).

**Figure 3:**
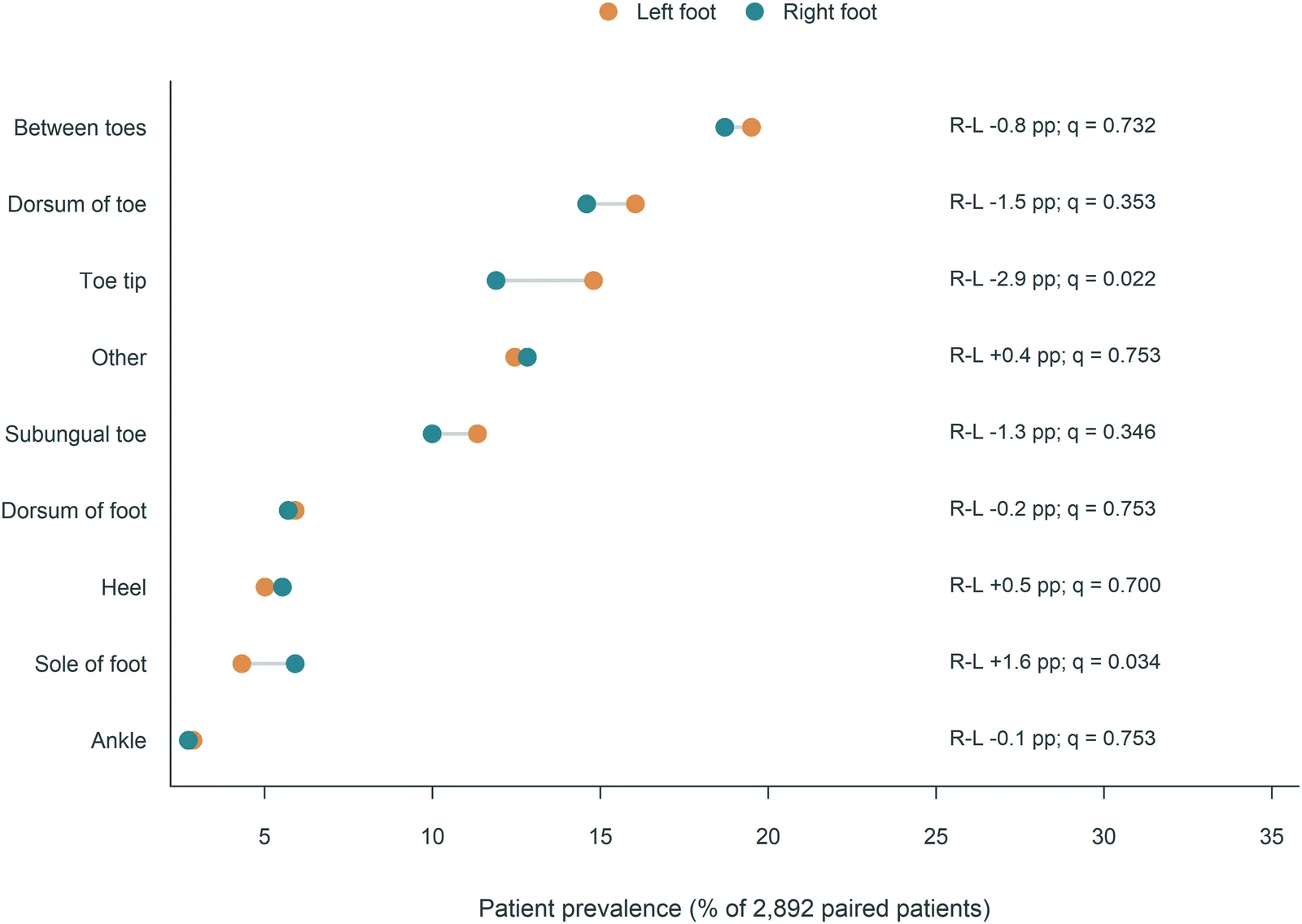
Paired comparison of recorded anatomical site involvement between left and right feet among 2,892 patients. Site indicators are non-exclusive and are derived from all locations recorded in the index record. R–L denotes right minus left in percentage points; q values are Benjamini–Hochberg-adjusted McNemar P values across nine grouped sites.

### 3.5 Associations of neurovascular pathology with recorded anatomical site involvement

Secondary cross-sectional analyses of recorded anatomical site involvement included 2,756 complete cases and used hospital-cluster-robust standard errors (**Figure 4**; **Supplementary Table S2**). Compared with patients with neither DPN nor LEAD, LEAD alone was associated with greater odds of dorsal-toe involvement (OR = 1.71, 95% CI 1.30–2.24; P<0.001; q=0.002). DPN alone was associated with heel involvement (OR = 1.83, 95% CI 1.22–2.76; P = 0.004; q=0.015). Coexisting DPN and LEAD was associated with toe-tip (OR = 2.14, 95% CI 1.36–3.37; P = 0.001; q=0.009) and subungual involvement (OR = 2.19, 95% CI 1.30–3.69; P = 0.003; q=0.015), and with lower odds of dorsum-of-foot involvement (OR = 0.47, 95% CI 0.32–0.69; P<0.001; q=0.002). DPN alone was not associated with sole involvement (OR = 0.96, 95% CI 0.61–1.49; P = 0.841; q=0.928). The associations were similar after additional adjustment for smoking and diabetes duration (**Supplementary Table S3**).

**Figure 4:**
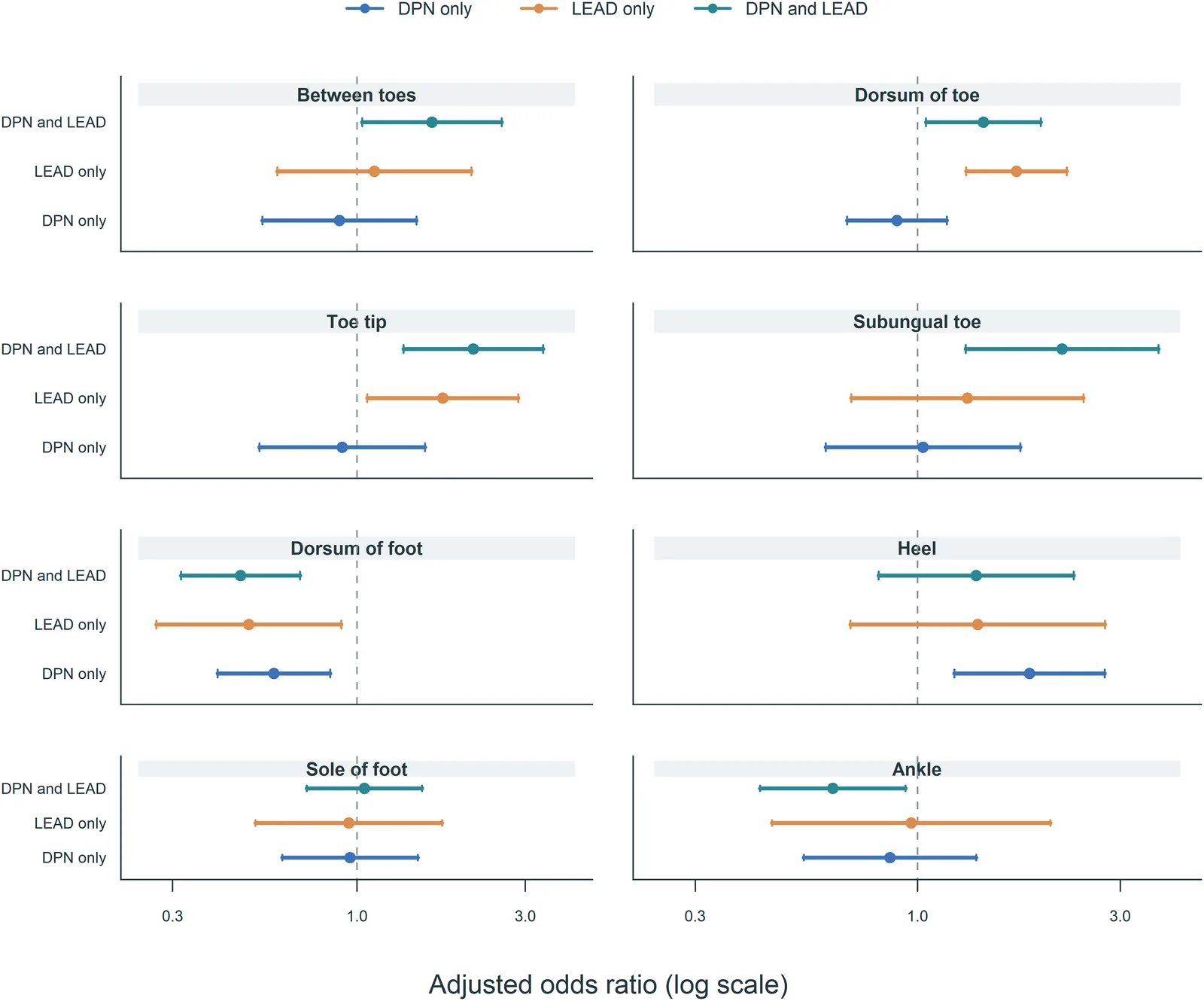
Adjusted cross-sectional associations between neurovascular status and recorded anatomical site involvement. Site indicators are non-exclusive and reflect recorded site involvement, not the sequence of onset. Odds ratios are adjusted for age and sex, with hospital-cluster-robust standard errors; the reference group is neither DPN nor LEAD. Bars show 95% confidence intervals; complete numerical results are provided in **Supplementary Table S2**.

## 4 Discussion

Based on multicenter data, this study used a 56-region foot classification to characterize the distribution of initial DFU sites. The detailed regions were summarized into eight prespecified anatomical categories plus an other category. Because patients could have more than one recorded site, the categories were non-exclusive, and the laterality, trigger-by-site, and neurovascular analyses used the same patient-level binary indicators. The detailed 56-region point distribution is retained as supplementary descriptive evidence, and the secondary analyses account for paired feet, hospital clustering, and multiple testing.

Between toes was the most frequently involved initial-site category, followed by dorsum of toe and toe tip, while the supplementary 56-region analysis identified the first-toe dorsum and the first-toe tip as the most frequent detailed points. Prompers et al. analyzed 1,229 diabetic foot ulcer patients from 14 European countries and found that 32% of ulcers were located on the dorsum or interdigital areas, although those locations were combined without further distinction (12). Direct comparison with previous studies is limited by differences in study populations, case definitions, and anatomical classification. The concentration of lesions around the toes, nail margins, and interdigital surfaces may reflect sustained pressure, shear, and footwear-related compression (13, 14). Other studies have categorized ulcers into broad regions such as forefoot, midfoot, and hindfoot, making it difficult to localize the initial lesion precisely (4, 15, 16).

Regarding ulcer precipitating factors, accidental injuries (32.2%) and footwear-related injuries (25.4%) were the most frequent categories. Unidentified causes accounted for 16.0%, potentially reflecting sensory loss that prevents recognition of minor trauma. Personal behaviors (15.3%), including improper nail trimming and self-treatment of calluses, also represented a substantial proportion. This is consistent with findings by Reiber, who noted that sensory loss may delay recognition of minor trauma and medical care (17). Chin et al. emphasized the association between poor self-care behaviors and diabetic foot ulcer risk (18), and Jones et al. identified inappropriate footwear as a common preventable precipitating factor (19). These findings point to potential targets for foot-care education, such as footwear selection and nail and callus care, which warrant evaluation in intervention studies.

Paired analyses identified two modest side differences: sole involvement was 1.59 percentage points more frequent on the right, whereas toe-tip involvement was 2.90 percentage points less frequent on the right. Both remained significant after FDR correction and suggest that side-specific patterns deserve attention during foot examination. Although gait asymmetry and limb dominance may contribute to laterality (20–22), the cross-sectional data do not establish a biomechanical mechanism, and the observed differences should be interpreted as site-specific descriptive associations.

Secondary neurovascular associations with recorded site involvement were site specific. LEAD alone was associated with greater odds of dorsal-toe involvement, while DPN alone was associated with heel involvement rather than sole involvement. Coexisting DPN and LEAD was associated with toe-tip and subungual involvement. These results partly support the clinical distinction between neuropathic pressure-related injury and distal ischemic injury (13), but they also show that broad mechanistic assumptions should not replace site-specific estimates. The inverse association between coexisting DPN and LEAD and dorsum-of-foot involvement requires cautious interpretation and external validation.

## 5 Study limitations

Although this study collected multicenter data from 77 hospitals, several limitations remain. First, hospitals were recruited through convenience sampling, which introduces hospital-based selection bias and limits representativeness beyond the study sample. Second, in patients with multiple recorded sites, lesions at different sites may develop concurrently or within a short interval; therefore, all recorded sites were classified as initial sites. Third, anatomical, DPN, LEAD, and precipitating-factor assessments were performed in routine practice without central adjudication, so between-center variation and misclassification are possible. Fourth, residual confounding by unmeasured factors is possible. Finally, the cross-sectional design precludes causal inference and longitudinal assessment. Prospective validation with standardized site adjudication is warranted.

## Data Availability

The raw data supporting the conclusions of this article will be made available by the authors, without undue reservation.

## References

[1] Tomic D Shaw JE Magliano DJ . The burden and risks of emerging complications of diabetes mellitus. Nat Rev Endocrinol. (2022) 18:525–39. doi: 10.1038/s41574-022-00690-7 35668219 PMC9169030

[2] Armstrong DG Boulton AJM Bus SA . Diabetic foot ulcers and their recurrence. N Engl J Med. (2017) 376:2367–75. doi: 10.1056/NEJMra1615439 28614678

[3] Zhang P Lu J Jing Y Tang S Zhu D Bi Y . Global epidemiology of diabetic foot ulceration: A systematic review and meta-analysis †. Ann Med. (2017) 49:106–16. doi: 10.1080/07853890.2016.1231932 27585063

[4] Jiang Y Wang X Xia L Fu X Xu Z Ran X . A cohort study of diabetic patients and diabetic foot ulceration patients in China. Wound Repair Regen: Off Publ Wound Heal Soc [and] Eur Tissue Repair Soc. (2015) 23:222–30. doi: 10.1111/wrr.12263 25682850

[5] Wang A Xu Z Mu Y Ji L . Clinical characteristics and medical costs in patients with diabetic amputation and nondiabetic patients with nonacute amputation in central urban hospitals in China. Int J Low Extrem Wounds. (2014) 13:17–21. doi: 10.1177/1534734614521235 24659623

[6] Armstrong DG Tan T-W Boulton AJM Bus SA . Diabetic foot ulcers. Jama. (2023) 330:62–75. doi: 10.1001/jama.2023.10578 37395769 PMC10723802

[7] Bus SA Sacco ICN Monteiro-Soares M Raspovic A Paton J Rasmussen A . Guidelines on the prevention of foot ulcers in persons with diabetes (IWGDF 2023 update). Diabetes Metab Res Rev. (2024) 40:e3651. doi: 10.1002/dmrr.3651 37302121

[8] Miller TA Campbell JH Bloom N Wurdeman SR . Racial disparities in health care with timing to amputation following diabetic foot ulcer. Diabetes Care. (2022) 45:2336–41. doi: 10.2337/dc21-2693 36069831 PMC9862414

[9] Schmidt BM Huang Y Banerjee M Hayek SS Pop-Busui R . Residential address amplifies health disparities and risk of infection in individuals with diabetic foot ulcers. Diabetes Care. (2024) 47:508–15. doi: 10.2337/dc23-1787 38241187 PMC10909679

[10] Alberti KG Zimmet PZ . Definition, diagnosis and classification of diabetes mellitus and its complications. Part 1: Diagnosis and classification of diabetes mellitus provisional report of a WHO consultation. Diabetes Med. (1998) 15:539–53. doi: 10.1002/(SICI)1096-9136(199807)15:7&lt;539::AID-DIA668>3.0.CO;2-S 9686693

[11] van Netten JJ Bus SA Apelqvist J Chen P Chuter V Fitridge R . Definitions and criteria for diabetes-related foot disease (IWGDF 2023 update). Diabetes Metab Res Rev. (2024) 40:e3654. doi: 10.1002/dmrr.3654 37186781

[12] Prompers L Huijberts M Apelqvist J Jude E Piaggesi A Bakker K . High prevalence of ischaemia, infection and serious comorbidity in patients with diabetic foot disease in europe. Baseline results from the eurodiale study. Diabetologia. (2007) 50:18–25. doi: 10.1007/s00125-006-0491-1 17093942

[13] Edmonds M . Diabetic foot ulcers: Practical treatment recommendations. Drugs. (2006) 66:913–29. doi: 10.2165/00003495-200666070-00003 16740006

[14] Bowling FL Reeves ND Boulton AJ . Gait-related strategies for the prevention of plantar ulcer development in the high risk foot. Curr Diabetes Rev. (2011) 7:159–63. doi: 10.2174/157339911795843159 21521160

[15] Oyibo SO Jude EB Tarawneh I Nguyen HC Armstrong DG Harkless LB . The effects of ulcer size and site, patient’s age, sex and type and duration of diabetes on the outcome of diabetic foot ulcers. Diabetes Med. (2001) 18:133–8. doi: 10.1046/j.1464-5491.2001.00422.x 11251677

[16] Pickwell KM Siersma VD Kars M Holstein PE Schaper NC on behalf of the Eurodiale consortium . Diabetic foot disease: Impact of ulcer location on ulcer healing. Diabetes Metab Res Rev. (2013) 29:377–83. doi: 10.1002/dmrr.2400 23390115

[17] Reiber GE Vileikyte L Boyko EJ del Aguila M Smith DG Lavery LA . Causal pathways for incident lower-extremity ulcers in patients with diabetes from two settings. Diabetes Care. (1999) 22:157–62. doi: 10.2337/diacare.22.1.157 10333919

[18] Chin Y-F Liang J Wang W-S Hsu B-S Huang T-T . The role of foot self-care behavior on developing foot ulcers in diabetic patients with peripheral neuropathy: A prospective study. Int J Nurs Stud. (2014) 51:1568–74. doi: 10.1016/j.ijnurstu.2014.05.001 24866324

[19] Jones PJ Armstrong DG Frykberg R Davies M Rowlands AV . Footwear fit as a causal factor in diabetes‐related foot ulceration: A systematic review. Diabetes Med. (2024) 41:e15407. doi: 10.1111/dme.15407 39012919

[20] Viteckova S Kutilek P Svoboda Z Krupicka R Kauler J Szabo Z . Gait symmetry measures: A review of current and prospective methods. BioMed Signal Process Control. (2018) 42:89–100. doi: 10.1016/j.bspc.2018.01.01342574925

[21] Sadeghi H Allard P Prince F Labelle H . Symmetry and limb dominance in able-bodied gait: A review. Gait Posture. (2000) 12:34–45. doi: 10.1016/S0966-6362(00)00070-9 10996295

[22] Kernozek TW Greany JF Heizler C . Plantar loading asymmetry in American Indians with diabetes and peripheral neuropathy, with diabetes only, and without diabetes. J Am Podiatr Med Assoc. (2013) 103:106–12. doi: 10.7547/1030106 23536500

