# Supplementary material for: Distribution characteristics of initial lesion sites for diabetic foot ulcers in China: a national multicenter survey
Source: Front Endocrinol (Lausanne). 2026 Oct 21;17:1983798. doi: 10.3389/fendo.2026.1983798 (PMC13730970; doi:10.3389/fendo.2026.1983798)
Supplement: Supplementary file 1 [file SupplementaryFile1.docx]

Supplementary Material

# 1 Supplementary Figures

**Supplementary Figure 1.** Frequency distribution across all 56 detailed anatomical locations by left foot, right foot, and overall. Numbers are location-point counts and colors represent percentages of side-specific or overall location points.

**Supplementary Figure 2.** Patient-level prevalence of grouped anatomical locations by precipitating-factor category among 2,892 patients. Cell values are percentages within trigger categories; column q values are Benjamini–Hochberg-adjusted P values from Pearson χ² global association tests across the nine grouped anatomical sites.
