# Supplementary material for: Distribution characteristics of initial lesion sites for diabetic foot ulcers in China: a national multicenter survey
Source: Front Endocrinol (Lausanne). 2026 Oct 21;17:1983798. doi: 10.3389/fendo.2026.1983798 (PMC13730970; doi:10.3389/fendo.2026.1983798)
Supplement: Supplementary file 2 [file Table1.docx]

**Supplementary Table S1.** Fifty-six-region anatomical classification used in Figure 1 and the corresponding grouped anatomical categories. Grouped categories were assigned from the recorded Chinese anatomical names using the same rules as the patient-level initial-site analysis.

| **Position** | **View** | **Anatomical region** | **Grouped category** |
| --- | --- | --- | --- |
| 1 | Dorsal (A) | First toe, subungual nail margin | Subungual toe |
| 2 | Dorsal (A) | First toe, dorsum of toe | Dorsum of toe |
| 3 | Dorsal (A) | First toe, tip | Toe tip |
| 4 | Dorsal (A) | First toe, lateral side | Between toes |
| 5 | Dorsal (A) | Second toe, subungual nail margin | Subungual toe |
| 6 | Dorsal (A) | Second toe, dorsum of toe | Dorsum of toe |
| 7 | Dorsal (A) | Second toe, tip | Toe tip |
| 8 | Dorsal (A) | Second toe, lateral side | Between toes |
| 9 | Dorsal (A) | Third toe, subungual nail margin | Subungual toe |
| 10 | Dorsal (A) | Third toe, dorsum of toe | Dorsum of toe |
| 11 | Dorsal (A) | Third toe, tip | Toe tip |
| 12 | Dorsal (A) | Third toe, lateral side | Between toes |
| 13 | Dorsal (A) | Fourth toe, subungual nail margin | Subungual toe |
| 14 | Dorsal (A) | Fourth toe, dorsum of toe | Dorsum of toe |
| 15 | Dorsal (A) | Fourth toe, tip | Toe tip |
| 16 | Dorsal (A) | Fourth toe, lateral side | Between toes |
| 17 | Dorsal (A) | Fifth toe, subungual nail margin | Subungual toe |
| 18 | Dorsal (A) | Fifth toe, dorsum of toe | Dorsum of toe |
| 19 | Dorsal (A) | Fifth toe, tip | Toe tip |
| 20 | Dorsal (A) | Fifth toe, lateral side | Between toes |
| 21 | Dorsal (A) | Dorsum of foot | Dorsum of foot |
| 22 | Dorsal (A) | Lateral side of fifth metatarsal | Other |
| 23 | Dorsal (A) | Lateral malleolus | Ankle |
| 24 | Dorsal (A) | Lateral heel | Heel |
| 25 | Dorsal (A) | Lateral midfoot | Other |
| 26 | Plantar (B) | First toe distal toe pad | Other |
| 27 | Plantar (B) | First toe interphalangeal joint, plantar aspect | Other |
| 28 | Plantar (B) | First toe medial side | Between toes |
| 29 | Plantar (B) | Second toe distal toe pad | Other |
| 30 | Plantar (B) | Second toe proximal interphalangeal joint, plantar aspect | Other |
| 31 | Plantar (B) | Second toe medial side | Between toes |
| 32 | Plantar (B) | Third toe distal toe pad | Other |
| 33 | Plantar (B) | Third toe proximal interphalangeal joint, plantar aspect | Other |
| 34 | Plantar (B) | Third toe medial side | Between toes |
| 35 | Plantar (B) | Fourth toe distal toe pad | Other |
| 36 | Plantar (B) | Fourth toe proximal interphalangeal joint, plantar aspect | Other |
| 37 | Plantar (B) | Fourth toe medial side | Between toes |
| 38 | Plantar (B) | Fifth toe distal toe pad | Other |
| 39 | Plantar (B) | Fifth toe proximal interphalangeal joint, plantar aspect | Other |
| 40 | Plantar (B) | Fifth toe medial side | Between toes |
| 41 | Plantar (B) | First and second toe interdigital skin | Between toes |
| 42 | Plantar (B) | Second and third toe interdigital skin | Between toes |
| 43 | Plantar (B) | Third and fourth toe interdigital skin | Between toes |
| 44 | Plantar (B) | Fourth and fifth toe interdigital skin | Between toes |
| 45 | Plantar (B) | First metatarsal plantar | Sole of foot |
| 46 | Plantar (B) | Second metatarsal plantar | Sole of foot |
| 47 | Plantar (B) | Third metatarsal plantar | Sole of foot |
| 48 | Plantar (B) | Fourth metatarsal plantar | Sole of foot |
| 49 | Plantar (B) | Fifth metatarsal plantar | Sole of foot |
| 50 | Plantar (B) | First metatarsal medial side | Other |
| 51 | Plantar (B) | Central sole | Sole of foot |
| 52 | Plantar (B) | Medial malleolus | Ankle |
| 53 | Plantar (B) | Medial heel | Heel |
| 54 | Plantar (B) | Plantar heel | Heel |
| 55 | Plantar (B) | Posterior heel | Heel |
| 56 | Plantar (B) | Medial midfoot | Other |

*Notes: Each of the 56 regions is assigned to one grouped category. Distal toe pads and plantar interphalangeal joints were grouped as Other rather than Sole of foot. Between toes comprises the interdigital skin and the medial and lateral surfaces of the toes.*
