# Supplementary material for: Distribution characteristics of initial lesion sites for diabetic foot ulcers in China: a national multicenter survey
Source: Front Endocrinol (Lausanne). 2026 Oct 21;17:1983798. doi: 10.3389/fendo.2026.1983798 (PMC13730970; doi:10.3389/fendo.2026.1983798)
Supplement: Supplementary file 3 [file Table2.docx]

**Supplementary Table S2. Adjusted associations between neurovascular status and recorded anatomical site involvement**

*Age- and sex-adjusted patient-level logistic regression with hospital-cluster-robust standard errors. Reference category: neither DPN nor LEAD.*

| **Anatomical site** | **Neurovascular status** | **Adjusted OR** | **95% CI** | **P value** | **FDR-adjusted q value** | **Site events / model n** |
| --- | --- | --- | --- | --- | --- | --- |
| Dorsum of toe | DPN only | 0.89 | 0.68–1.17 | 0.419 | 0.670 | 852/2,756 |
|  | LEAD only | 1.71 | 1.30–2.24 | <0.001 | 0.002 | 852/2,756 |
|  | DPN and LEAD | 1.43 | 1.05–1.95 | 0.025 | 0.062 | 852/2,756 |
| Between toes | DPN only | 0.89 | 0.54–1.47 | 0.656 | 0.919 | 1,051/2,756 |
|  | LEAD only | 1.12 | 0.59–2.11 | 0.725 | 0.919 | 1,051/2,756 |
|  | DPN and LEAD | 1.63 | 1.03–2.57 | 0.035 | 0.077 | 1,051/2,756 |
| Sole of foot | DPN only | 0.96 | 0.61–1.49 | 0.841 | 0.928 | 277/2,756 |
|  | LEAD only | 0.95 | 0.52–1.74 | 0.864 | 0.928 | 277/2,756 |
|  | DPN and LEAD | 1.05 | 0.72–1.53 | 0.800 | 0.928 | 277/2,756 |
| Dorsum of foot | DPN only | 0.58 | 0.40–0.84 | 0.004 | 0.015 | 321/2,756 |
|  | LEAD only | 0.49 | 0.27–0.90 | 0.022 | 0.062 | 321/2,756 |
|  | DPN and LEAD | 0.47 | 0.32–0.69 | <0.001 | 0.002 | 321/2,756 |
| Toe tip | DPN only | 0.91 | 0.53–1.56 | 0.727 | 0.919 | 741/2,756 |
|  | LEAD only | 1.75 | 1.07–2.87 | 0.026 | 0.062 | 741/2,756 |
|  | DPN and LEAD | 2.14 | 1.36–3.37 | 0.001 | 0.009 | 741/2,756 |
| Subungual toe | DPN only | 1.03 | 0.61–1.75 | 0.912 | 0.928 | 588/2,756 |
|  | LEAD only | 1.31 | 0.70–2.46 | 0.400 | 0.670 | 588/2,756 |
|  | DPN and LEAD | 2.19 | 1.30–3.69 | 0.003 | 0.015 | 588/2,756 |
| Heel | DPN only | 1.83 | 1.22–2.76 | 0.004 | 0.015 | 278/2,756 |
|  | LEAD only | 1.39 | 0.70–2.76 | 0.354 | 0.653 | 278/2,756 |
|  | DPN and LEAD | 1.37 | 0.81–2.33 | 0.239 | 0.477 | 278/2,756 |
| Ankle | DPN only | 0.86 | 0.54–1.37 | 0.531 | 0.796 | 153/2,756 |
|  | LEAD only | 0.97 | 0.45–2.05 | 0.928 | 0.928 | 153/2,756 |
|  | DPN and LEAD | 0.63 | 0.43–0.94 | 0.022 | 0.062 | 153/2,756 |

Notes: Outcomes were non-exclusive patient-level indicators derived from all locations recorded in the index record. Adjusted odds ratios compare each neurovascular category with neither DPN nor LEAD. FDR-adjusted q values use the Benjamini–Hochberg procedure across all 24 coefficients. DPN, diabetic peripheral neuropathy; LEAD, lower extremity arterial disease; OR, odds ratio; CI, confidence interval.
