# Supplementary material for: Distribution characteristics of initial lesion sites for diabetic foot ulcers in China: a national multicenter survey
Source: Front Endocrinol (Lausanne). 2026 Oct 21;17:1983798. doi: 10.3389/fendo.2026.1983798 (PMC13730970; doi:10.3389/fendo.2026.1983798)
Supplement: Supplementary file 4 [file Table3.docx]

**Supplementary Table S3. Sensitivity analysis with additional adjustment for smoking and diabetes duration**

Patient-level logistic regression with hospital-cluster-robust standard errors. Reference category: neither DPN nor LEAD. Main model: adjusted for age and sex (complete cases, n = 2,756). Extended model: additionally adjusted for smoking and diabetes duration (complete cases, n = 2,441).

Notes: Outcomes were non-exclusive patient-level indicators derived from all locations recorded in the index record. FDR-adjusted q values were calculated across the 24 estimates within each model (Benjamini–Hochberg). OR, odds ratio; CI, confidence interval; DPN, diabetic peripheral neuropathy; LEAD, lower extremity arterial disease.

| **Anatomical site** | **Neurovascular status** | **Main model OR (95% CI)** | **P** | **q** | **Extended model OR (95% CI)** | **P** | **q** | **Events / n (main)** | **Events / n (extended)** |
| --- | --- | --- | --- | --- | --- | --- | --- | --- | --- |
| Between toes | DPN only | 0.89 (0.54–1.47) | 0.656 | 0.919 | 0.92 (0.55–1.55) | 0.760 | 0.829 | 1,051/2,756 | 958/2,441 |
|  | LEAD only | 1.12 (0.59–2.11) | 0.725 | 0.919 | 1.33 (0.75–2.36) | 0.337 | 0.578 | 1,051/2,756 | 958/2,441 |
|  | DPN and LEAD | 1.63 (1.03–2.57) | 0.035 | 0.077 | 1.92 (1.29–2.86) | 0.001 | 0.005 | 1,051/2,756 | 958/2,441 |
| Dorsum of toe | DPN only | 0.89 (0.68–1.17) | 0.419 | 0.670 | 0.93 (0.67–1.27) | 0.632 | 0.788 | 852/2,756 | 773/2,441 |
|  | LEAD only | 1.71 (1.30–2.24) | <0.001 | 0.002 | 1.84 (1.40–2.42) | <0.001 | <0.001 | 852/2,756 | 773/2,441 |
|  | DPN and LEAD | 1.43 (1.05–1.95) | 0.025 | 0.062 | 1.52 (1.13–2.04) | 0.006 | 0.017 | 852/2,756 | 773/2,441 |
| Toe tip | DPN only | 0.91 (0.53–1.56) | 0.727 | 0.919 | 0.91 (0.52–1.59) | 0.741 | 0.829 | 741/2,756 | 678/2,441 |
|  | LEAD only | 1.75 (1.07–2.87) | 0.026 | 0.062 | 1.87 (1.09–3.21) | 0.024 | 0.055 | 741/2,756 | 678/2,441 |
|  | DPN and LEAD | 2.14 (1.36–3.37) | 0.001 | 0.009 | 2.25 (1.43–3.54) | <0.001 | 0.003 | 741/2,756 | 678/2,441 |
| Subungual toe | DPN only | 1.03 (0.61–1.75) | 0.912 | 0.928 | 0.96 (0.55–1.66) | 0.875 | 0.913 | 588/2,756 | 530/2,441 |
|  | LEAD only | 1.31 (0.70–2.46) | 0.400 | 0.670 | 1.43 (0.70–2.92) | 0.321 | 0.578 | 588/2,756 | 530/2,441 |
|  | DPN and LEAD | 2.19 (1.30–3.69) | 0.003 | 0.015 | 2.31 (1.38–3.86) | 0.001 | 0.005 | 588/2,756 | 530/2,441 |
| Dorsum of foot | DPN only | 0.58 (0.40–0.84) | 0.004 | 0.015 | 0.59 (0.43–0.80) | <0.001 | 0.004 | 321/2,756 | 300/2,441 |
|  | LEAD only | 0.49 (0.27–0.90) | 0.022 | 0.062 | 0.56 (0.34–0.93) | 0.025 | 0.055 | 321/2,756 | 300/2,441 |
|  | DPN and LEAD | 0.47 (0.32–0.69) | <0.001 | 0.002 | 0.51 (0.35–0.74) | <0.001 | 0.003 | 321/2,756 | 300/2,441 |
| Heel | DPN only | 1.83 (1.22–2.76) | 0.004 | 0.015 | 2.02 (1.32–3.09) | 0.001 | 0.005 | 278/2,756 | 242/2,441 |
|  | LEAD only | 1.39 (0.70–2.76) | 0.354 | 0.653 | 1.20 (0.54–2.63) | 0.657 | 0.788 | 278/2,756 | 242/2,441 |
|  | DPN and LEAD | 1.37 (0.81–2.33) | 0.239 | 0.477 | 1.36 (0.83–2.25) | 0.225 | 0.451 | 278/2,756 | 242/2,441 |
| Sole of foot | DPN only | 0.96 (0.61–1.49) | 0.841 | 0.928 | 0.82 (0.50–1.34) | 0.422 | 0.633 | 277/2,756 | 252/2,441 |
|  | LEAD only | 0.95 (0.52–1.74) | 0.864 | 0.928 | 0.82 (0.41–1.64) | 0.579 | 0.788 | 277/2,756 | 252/2,441 |
|  | DPN and LEAD | 1.05 (0.72–1.53) | 0.800 | 0.928 | 0.90 (0.59–1.35) | 0.600 | 0.788 | 277/2,756 | 252/2,441 |
| Ankle | DPN only | 0.86 (0.54–1.37) | 0.531 | 0.796 | 0.81 (0.49–1.34) | 0.421 | 0.633 | 153/2,756 | 132/2,441 |
|  | LEAD only | 0.97 (0.45–2.05) | 0.928 | 0.928 | 1.00 (0.41–2.42) | 0.994 | 0.994 | 153/2,756 | 132/2,441 |
|  | DPN and LEAD | 0.63 (0.43–0.94) | 0.022 | 0.062 | 0.56 (0.36–0.86) | 0.009 | 0.023 | 153/2,756 | 132/2,441 |
